# Correlation of the Thermal Conductivity and Mechanical Properties in Hybrid Filler Systems of Thermosets

**DOI:** 10.3390/polym17141924

**Published:** 2025-07-12

**Authors:** Uta Rösel, Dietmar Drummer

**Affiliations:** Institute of Polymer Technology, Friedrich-Alexander-University Erlangen-Nuremberg, 91058 Erlangen, Germany; dietmar.drummer@fau.de

**Keywords:** epoxy resin, two-component fillers, thermal conductivity, mechanical properties

## Abstract

Thermal management reveals an increasing importance due to the changing demands in terms of the compactness and the performance of electronic devise. Polymers in general and thermosets specifically depict a low thermal conductivity, where filler systems are needed to improve performance and make polymers suitable for certain applications. So far, different influencing factors in terms of improving the thermal conductivity in thermosets, mainly through the use of single-filler systems, have been investigated in. To some extent, hybrid filler systems have been examined as well; however, the behavior itself in terms of the thermal conductivity as well as the mechanical properties is rather unknown. In terms of the applications, it is essential to understand the correlation between the thermal conductivity and the mechanical properties as this is the fundamental requirement to realize a proper dimensioning of samples in applications. Therefore, this paper investigates hybrid filler systems based on boron nitride (BN) and three different second fillers with varying ratios and in terms of both the improvement of the thermal conductivity and the mechanical properties. Copper (Cu) was shown to reach the best compromise within the hybrid materials. Furthermore, criteria of an improved thermal flow path and sufficient mechanical properties have been stated in general.

## 1. Introduction

### 1.1. Application Fields and Possible Extensions

As modern electronic devices like power electronics and electric motors become more compact and performant, effective thermal management is becoming increasingly important for the design of samples in these application areas. This is in conjunction with the increased heat generated by larger power devices and samples, which permit higher operating temperatures, such as silicon carbide chips, for instance. Over the past two decades, the potential operating temperature of silicon carbide semiconductors has risen from 90 °C [1] to 250 °C [2]. The required heat resistance for applications like electronics or electric motor encapsulation results in the usage of thermosets, primarily epoxy resin [3].

The materials must provide a high thermal conductivity and electrical insulation. Since polymers typically exhibit low electric and thermal conductivity, fillers are required to meet both of these demands. Due to their different viscosity behaviors and lower values compared to thermoplastics, thermosets show potential as better filler contents compared to thermoplastics.

### 1.2. Mechanisms of Thermal Conductivity of Thermoset-Based Compounds

Heat transfer in polymers occurs primarily through the propagation of elastic waves along a single macromolecule via its covalent bonds [4]. Additionally, secondary valence bonds such as van der Waals forces facilitate heat transfer along molecules [5]. The transport within a molecule is more effective than the inter-molecular method. The thermal conductivity rises along with the molecular mass, as the number of chain ends—and consequently the number of van der Waals bonds—decreases. Ref. [6] states that the thermal conductivity is restricted in relation to the increasing molecular mass, which relies on the chemical structure of the polymer [6]. Further, the heat transfer relies on an energy balance, which includes conductive and convective parts. It is likely that the convective terms are not discussed, especially in models of the heat transfer on thermoset-based composites [7].

Filler systems are needed to increase the potential thermal conductivity of polymers. The upper range of thermal conductivity for filled epoxy resin lies between 10 and 20 W∙m^−1^∙K^−1^. In [8], a thermal conductivity of 10.98 W∙m^−1^∙K^−1^ was demonstrated using an aluminum nitride filler system with a filler grade of 60 vol.-% and silane surface modification in epoxy resin. Through the combination of various anisotropic boron nitride particles, ref. [9] attained a thermal conductivity of 19.0 W∙m^−1^∙K^−1^, also based on the epoxy. Unfilled thermosets reach only values between 0.1 and 1 W∙m^−1^∙K^−1^, which underlines the need for fillers in thermosets in order to reach the required thermal conductivity [10]. To increase the thermal conductivity of filled thermosets, the interfacial thermal resistance (ITR) has to be taken into account, after its diminishing effect on the thermal transfer. The ITR can be divided into the matrix–filler interface and the filler–filler interface. The ITR should be as low as possible to improve the thermal conductivity [11]. This can be realized by an in situ growth of hetero-structured fillers [12], a surface functionalization of the fillers [13], an alignment of fillers [14] or a bridging of the fillers [15]. The in situ strategy is only effective in terms of the reduced filler–filler interfaces [10]. However, the orientation of fillers shall be avoided in terms of some applications, according to [10].

Furthermore, the intrinsic thermal conductivity of at least epoxy-based resins can be increased by implementing a liquid-crystal structure, and thereby a molecular orientation and stiffness on a molecular level [16]. This is not part of the following manuscript and will therefore not be portrayed in detail.

### 1.3. Influencing Factors on the Thermal Conductivity of Filled Thermosets

To sustain electric isolating behavior along with thermal conductivity, mainly mineral or ceramic filler types are utilized [17]. Filler systems based on boron nitride (BN), zinc oxide (ZnO), or graphite are most commonly used to enhance the thermal conductivity of polymers. Boron nitride (BN)’s initial thermal conductivity is around tenfold greater than that of zinc oxide (ZnO), which is depicted in Section 2 in detail [18]. Graphite or, in general, carbon-based fillers combine the advantages of a high possible thermal conductivity and lightweight aspects [10]. In addition to the initial characteristics of the filler, several factors have been identified that affect the thermal conductivity of the compound. The factors, which have an impact on the thermal conductivity, are the matrix material, filler size, size distribution, and geometry, as stated in [18]. While the number of contact points is essential for thermal conductivity, the filler content is also significant. The proof provided in [19] demonstrated that a higher filler grade combined with a greater aspect ratio leads to a more significant enhancement of thermal conductivity than lower aspect ratios [19], due to longer conduction paths [20]. Platelet or rod-shaped fillers generally exhibit higher thermal conductivity than spherical ones [5]. These 2D filler geometries (platelets, sheets, flakes) reveal a large contact area at the filler–filler interface and with that, a high ITR. When the interstitial area is small, the overall ITR is reduced, which results in a high thermal conductivity [20]. Ref. [21] considers the shape of the filler as more important compared to the size of the filler in terms of the thermal conductivity, at least in terms of nano-scaled particles [21]. However, these geometries often result in anisotropic properties across the sample, with thermal conductivity being predominantly high in one direction, such as along the fiber axis. Further, filler shapes with smooth surfaces should be considered to improve the processability and reduce the friction between particles [20]. From the perspective of a high amount of contact points and extensive contact zones, larger particles are favored, particularly when they exhibit an orientation and thus a conductive route [6]. This positive effect of large filler sizes on the thermal conductivity goes along with the reduced specific surface area of the particles which reduce the filler–matrix interface and with that, the ITR [20]. However, ref. [20] proved that larger fillers may have a negative effect onto the thermal conductivity due to higher percentages of defect density in the compound. This has to be considered especially in the presence of excessive oxygen atoms in graphene layers [22].

It must be considered that size is just one factor in relation to the enhancement of thermal conductivity. Larger fillers, while they increase the number of contact points in cases of direct tangency, have a greater average distance between them compared to smaller fillers of the same filler grade. In that regard, it is possible that the thermal conductivity does not improve with the use of large fillers, since other aspects must also be considered. Moreover, hybrid systems that combine small and large particle sizes reduce the space between fillers, thereby enhancing thermal conductivity [23]. Further, single-filler systems shall be avoided in the future as high filler amounts are needed, which reduce the lightweight aspect and increase the brittle behavior. It can be assumed, according to [20], that hybrid filler systems with two or more fillers can increase the thermal conductivity in a higher amount compared to a single-filler system within the same or even lower filler grades due to the usage of synergistic effects [20]. It depends on the combination of the filler systems in hybrid systems, and whether the contact area is a point or a line; a line contact should be aspired for as the contact area is larger and the heat transfer is more likely across the filler–filler interface. Specificall, the combination of 0Ds (nanoparticles) and 1D (fiber, rods, tubes, wires) should be avoided in terms of increasing the thermal conductivity in hybrid filler systems [20]. Hybrid filler systems are based on different filler types and different geometries within these types. They are unlikely to be a combination of the same filler type with different geometries—as would be possible, for instance, in terms of copper spheres and platelets. However, hybrid filler systems often include more than one filler type. For example, the following hybrid filler systems have been investigated: carbon nanotubes with graphite nano-platelets [24], alumina particles with graphene [25], or boron nitride with graphene particles [15]. Graphene or carbon nanotubes are two commonly used filler types, which are often combined with larger fillers such as copper, aluminum, or aluminum oxide. With the main objective of increasing the thermal conductivity, which is based on an increasing number of contact points, the choice of the different filler types is made in consideration with different filler geometries and sizes in order to increase the contact points.

### 1.4. Aim of the Paper

The demands in power electronic devices lead to the request for a higher thermal conductivity within thermoset-based materials, which should be realized by a hybrid filler system. This approach reveals the opportunity to reduce the overall filler amount by using a synergistic effect between the fillers. Even though different influencing factors, mainly in terms of a single-filler system, have been investigated, the usage of synergistic effects and the possibility of a reduced filler amount are still unclear. Further, the investigations focus only on the thermal conductivity as a property, which should be optimized. Other characteristic behaviors such as the mechanical properties are unlikely to be analyzed. The mechanical properties, especially within the context of the thermal conductivity, are essential to know, as the dimensions of samples for applications rely on the correlation of both attributes. Therefore, the understanding of the interactions between both factors—thermal conductivity and mechanical properties—is highly important for constructions within applications.

Therefore, this paper investigates both the aspects of a high thermal conductivity and sufficient mechanical properties with the aim of realizing an adequate thermal flow path in terms of thermal conductivity. With respect to electronic devices, typical materials for encapsulation are epoxy resins, such as EP 3162 E (Raschig GmbH, Ludwigshafen, Germany), for instance. The manufacturer’s specification of this type of material was defined as an industrial standard in terms of the mechanical properties within this paper, which results in the following values: an E-Modulus E_t_ of at least 17.4 GPa, a tensile strength σ_m_ of 76.7 MPa, and an elongation at break ε_m_ of 0.45%.

The paper investigates three different hybrid filler systems, all based on boron nitride as the main filler type. The filler grade is kept constant and rather low with 40 vol.-%; further, the ratio of the two fillers is varied as 50:50 and 75:25. The subsequent filler systems include glass, copper and zinc oxide. After boron nitride revealed a rather small particle size, copper was chosen in terms of its large size and high thermal conductivity. Further, the spheres of copper lead to rather large spaces between each filler, which revealed an interrupted flow path of the thermal conductivity. To improve this flow path, the plate structure of boron nitride is expected to largely close these gaps. Glass was chosen as a reference material, where the impact on the thermal conductivity is negligible. To compare the geometrical impact of spheres, glass was chosen in the same geometry as the copper. However, the size of the glass and copper were different. The last filler is zinc oxide, which reveals a star geometry. Previous investigations proved that the star geometry is broken into mainly small fractures. This filler was therefore chosen to close the gaps between the boron nitride fillers, similarlt to what was intended with boron nitride and copper. Primarily, a material system shall be defined where both aspects reach the industrial standard, at least.

## 2. Materials and Methods

### 2.1. Materials

The matrix material within these experiments was an epoxy resin of the type Epoxidur EP 368/1 (Raschig GmbH, Ludwigshafen, Germany), which is a pre-mixture of resin, hardener, a catalyst, and carbon black pigments with the exact formulation being confidential. Table 1 depicts the specification of the matrix material in terms of the density δ, heat capacity c (based on our own measurements), and thermal conductivity λ (based on manufacturer specification).

Within the experiments, the main filler was defined as boron nitride (BN) of the type CFA 50M SFG (3M Deutschland GmbH, Neuss, Germany) which has a platelet structure. In addition, three different filler systems were added to analyze the possibility of improving the thermal conductivity λ without reducing the mechanical properties by using hybrid filler systems. Glass spheres (G) of the type 3000 CP 03 (Velox GmbH, Hamburg, Germany) were used as a reference additional filler with a thermal conductivity λ of 1 W∙m^−1^∙K^−1^, in comparison with copper spheres (Cu) of the type GK 0|50 (Schlenk Metallic Pigments GmbH, Roth, Germany). Further, zinc oxide (ZnO) of the type Silatherm Advanced 1438–800 (Quarzwerke GmbH, Frechen, Germany) was investigated as it has a very narrow particle size and a star-like geometry. Table 2 depicts the specifications of the filler systems in terms of the density δ, the particle size in terms of numerical and volumetric counting, the heat capacity c (based on our own measurements), and the thermal conductivity λ (based on manufacturer specification). Further, Table 3 shows the particle geometry based on images taken by a scanning electron microscope SEM of the type Gemini Ultra-Plus (Carl Zeiss AG, Oberkochen, Germany).

The filler grade was kept constant with 40 vol.-%; within the hybrid filler systems, the addition of glass (G), copper (Cu), or zinc oxide (ZnO) to boron nitride (BN) was realized in the ratios of 50:50 and 25:75. Further, test samples with the pure fillers (single-filler systems) were realized with the same filler grade of 40 vol.-% as a reference.

### 2.2. Fabrication of the Test Specimens

The production of the test specimens was realized by injection molding after the material was fabricated by a compounding process. The matrix and the filler systems were mixed manually at room temperature. The exact proportion of each component was controlled by a high-precision weighting device. Homogeneous and sufficient mixing was assured by an optical control. The compounds were produced in a twin-screw extruder (type: Kraus Maffei Berstorff ZSE 25Ax45D; KrausMaffei Group, Munich, Germany) with a constant rotational speed for the screw of 80 min^−1^ and a temperature range from 50 °C to 90 °C from the feeding zone to the nozzle. The material was cooled using a vibratory feeder (Volkmann Group, Soest, Germany), followed by pelletizing.

The injection molding machine (type: KM 80-380 CX DUR/03; Kraus Maffei Group, Munich, Germany) had a screw diameter of 30 mm. To save material, the samples for the characterization of the thermal diffusivity and the mechanical properties were kept constant with a plate structure in the dimensions of 60 × 60 × 2 [cm^3^]. The process parameters were kept as constant as possible within the different filler systems as shown in Table 4. The main adjustments took place in the feeding zone temperature and the mold temperature as well as slight changes in the heating time.

### 2.3. Characterization

#### 2.3.1. Differential Scanning Calorimetry (DSC) According to DIN EN ISO 11357

The temperature-dependent reaction kinetic of the material was characterized using differential scanning calorimetry (type: DSC 2500; TA instruments, New Castle, DE, USA) under a nitrogen atmosphere according to DIN EN ISO 11357 [26] in order to analyze the process conditions in both fabrication processes. Roughly 5 mg of each material system was heated with a rate of 10 K∙min^−1^ within the temperature range of 0 and 300 °C. The reaction turnover α was characterized based on this first heat cycle, where α is in the ratio of the specific enthalpy at the temperature level T_j_ (ΔH_j_) and the total specific enthalpy (ΔH_total;1_).

#### 2.3.2. Density ρ

Test parts with dimensions of 10 × 10 × 2 [cm^3^] were prepared from the middle of the fabricated sample to define the density. The preparation was realized through the usage of a water-cooled saw to ensure a reduced temperature impact. After the preparation, the dimensions of the test parts were measured by a digital caliper with a resolution of 0.01 mm and the weights were measures using a high-precision weighting device. With that, the density could be defined as the ratio of weight per volume. In addition, the theoretical density was calculated based on the density of the pure materials as depicted in Table 1 for the matrix material and as depicted in Table 2 for the filler systems. Further, the density is part of the calculation of the thermal conductivity λ, which is discussed in detail in Section 2.3.5.

#### 2.3.3. Specific Heat Capacity c

In addition to the density, the specific heat capacity c is needed to calculate the thermal conductivity λ. Therefore, the specific heat capacity c was measured at 25 °C using a C80 calorimeter (type: 3D-Calvet calorimeter; TA Instruments, New Castle, DE, USA). Ref. [27] already proved that the theoretical value of the specific heat capacity c calculated on the basis of the pure materials reveals a broad consensus with the measured values based on the compounds. Although the specific heat capacity c is temperature-dependent, the calculation within this paper is only held at room temperature as the application is expected to be in this temperature range as well.

#### 2.3.4. Thermal Diffusivity a According to DIN EN ISO 22007

With respect to possible applications within housings in power electronics, the directional-dependent thermal diffusivity a was only defined in the *z*-direction in the middle of the test sample (perpendicular to the flow direction and with the through plane). A nanoflash device (type: LFA447; Netzsch GmbH, Selb, Germany) according to DIN EN ISO 22007 [28] was applied at a temperature of 23 °C.

#### 2.3.5. Thermal Conductivity λ According to DIN EN ISO 22007

Once the thermal conductivity λ is more in alignment with the values in the literature, the results of the thermal diffusivity a (in Section 2.3.4) were converted through multiplication with the density *ρ* and the specific heat capacity c. In terms of the density *ρ*, the ideal values were used, in order to focus on the impact of the optimized process.

#### 2.3.6. Mechanical Properties According to DIN EN ISO 527

To analyze the mechanical properties, strips with dimensions of 15 × 60 × 2 [cm^3^] were prepared in the flow direction from the middle of the test samples. Again, a water-cooled saw was used to reduce the temperature impact. Afterwards, a tensile bar of the type A23 according to [29] was extracted using a CNC mill (POS GmbH & Co. KG, Rechberghausen, Germany). A universal tensile testing machine (type: 1464; ZwickRoell GmbH & Co. KG, Ulm, Germany) according to DIN EN ISO 527 [30] was utilized and the mechanical properties (stiffness represented by the E-Modulus E_t_, tensile strength σ_m_, and elongation at break ε_m_) were determined at 23 °C. The traverse speed was kept constant at the value of 0.5 mm∙min^−1^.

#### 2.3.7. Filler Distribution

To analyze the filler distribution, especially in terms of generating a thermal flow path along the fillers with ideally a high number of contact points, small strips with a width of 3 mm were prepared. These strips were taken in the flow direction from the middle of the gating system using a water-cooled saw and with that minimum temperature impact. The samples were embedded in cold-curing epoxy resin (type: Epofix; Struers GmbH, Ottensoos, Germany) and grinded as well as polished afterwards. In addition, a 10 nm layer of sprayed gold was applied. Images were taken by a scanning electron microscope (type: Gemini Ultra-Plus; Carl Zeiss AG, Oberkochen, Germany) with a magnification between 200 and 750, depending on the filler size.

## 3. Results and Discussion

### 3.1. Results of Differential Scanning Calorimetry (DSC) According to DIN EN ISO 11357

**Figure 1:**
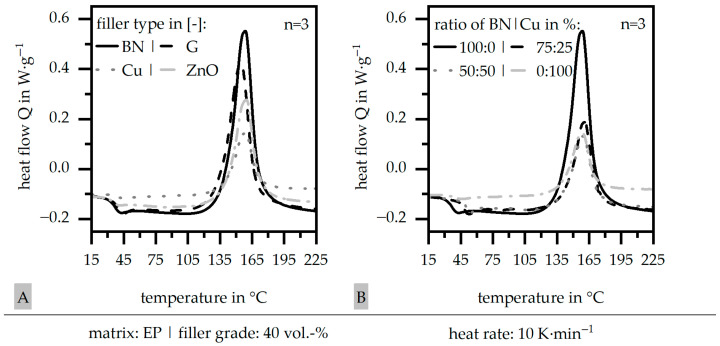
Heat flow Q relative to the temperature based on DSC measurements of the compound material relative to the filler system (single (**A**)) and exemplarily for boron nitride (BN) and copper (Cu) with varying ratios (50:50 or 75:25) (**B**) for a constant filler grade of 40 vol.-% in comparison to the pure resin.

The general route of the heat flow Q is portrayed in Figure 1A in terms of the four single fillers (boron nitride (BN), glass (G), copper (Cu), and zinc oxide (ZnO)) and exemplarily in Figure 1B in terms of boron nitride (BN) and copper (Cu) for a hybrid system relative to the different ratios and the pure materials. The peak temperature is slightly shifted to lower values in terms of glass (G) (and compared to the other single-filler systems). The specific enthalpy, which is required for the curing, is reduced in terms of glass (G) and mainly in terms of copper (Cu) and zinc oxide (ZnO) compared to boron nitride (BN). This could be reasoned in terms of the lower heat capacity c for copper (Cu) and zinc oxide (ZnO) compared to boron nitride (BN), although glass (G) has the same heat capacity c relative to boron nitride (BN). In general, the single-filler systems show similar behavior in terms of the curing. The hybrid filler systems follow mainly the route of copper (Cu) in this example, or, in general, the second filler system with boron nitride (BN). This leads to a reduced enthalpy impact, which is needed for the curing process. At this point, the authors cannot explain why the behavior of boron nitride (BN) is so suppressed even if the ratio is 75:25 in the hybrid system.

**Figure 2:**
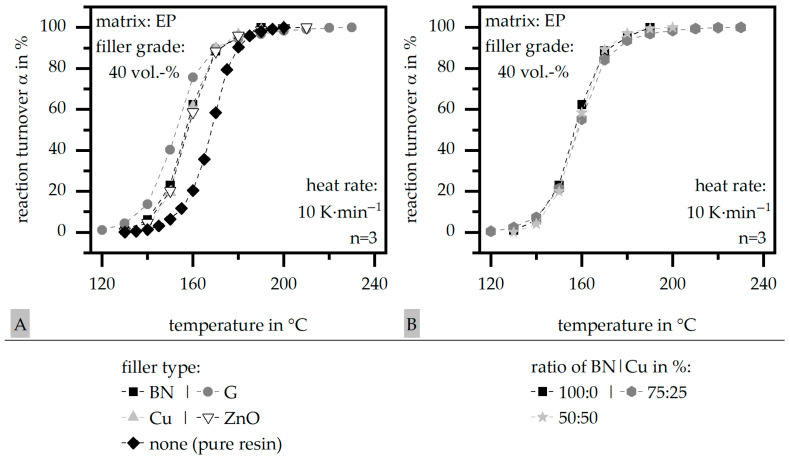
Reaction turnover α relative to the temperature based on DSC measurements of the compound material relative to the filler system (single (**A**)) and exemplarily for boron nitride (BN) and copper (Cu) with varying ratios (50:50 or 75:25) (**B**) for a constant filler grade of 40 vol.-% in comparison to the pure resin.

Figure 2 depicts the reaction turnover α relative to the temperature in terms of the single-filler systems in Figure 2A and in terms of the hybrid systems exemplarily for boron nitride (BN) and Cu in Figure 2B. Further, the reaction turnover α is compared to the pure matrix material in terms of the single fillers. The reaction turnover α is shifted to lower temperatures for the single-filler systems compared to the pure resin, where glass (G) depicts the highest shift with the lowest thermal conductivity λ. The change in the ratio within the hybrid system boron nitride (BN) and copper (Cu) does not effect the reaction turnover α relative to the temperature.

### 3.2. Results of Density ρ

**Figure 3:**
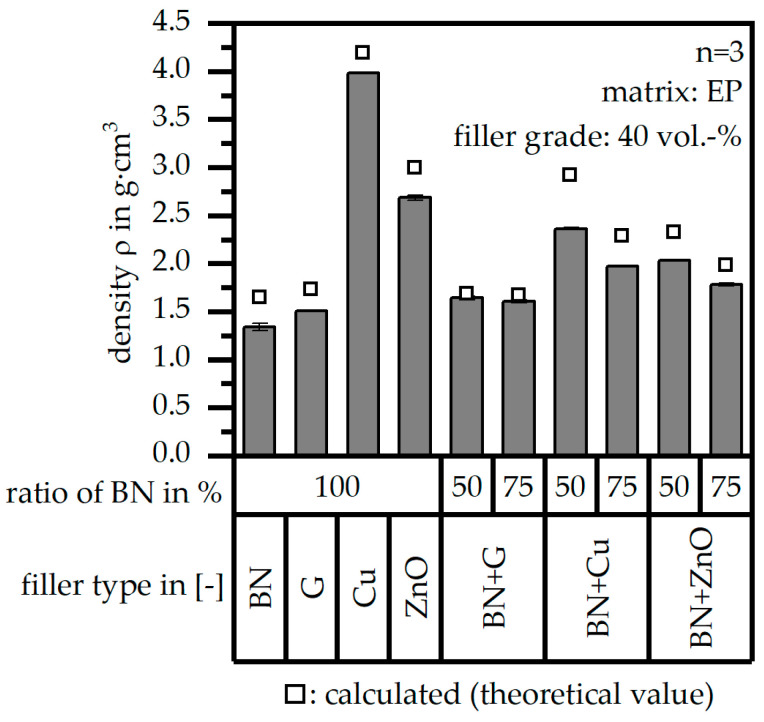
Density *ρ* of the compound material relative to the filler system (single or hybrid) with varying ratios (50:50 or 75:25) and a constant filler grade of 40 vol.-% in comparison to the theoretical (calculated) values.

Figure 3 shows the density ρ of the test samples for the single-filler and the hybrid filler systems (in terms of the two ratios) in comparison to the theoretical value, which was calculated based on the density ρ of the pure materials. The theoretical values are slightly higher compared to the calculated ones. This indicates further improvement within the fabrication process to reach values nearer to the theoretical value.

### 3.3. Results of Specific Heat Capacity c

**Figure 4:**
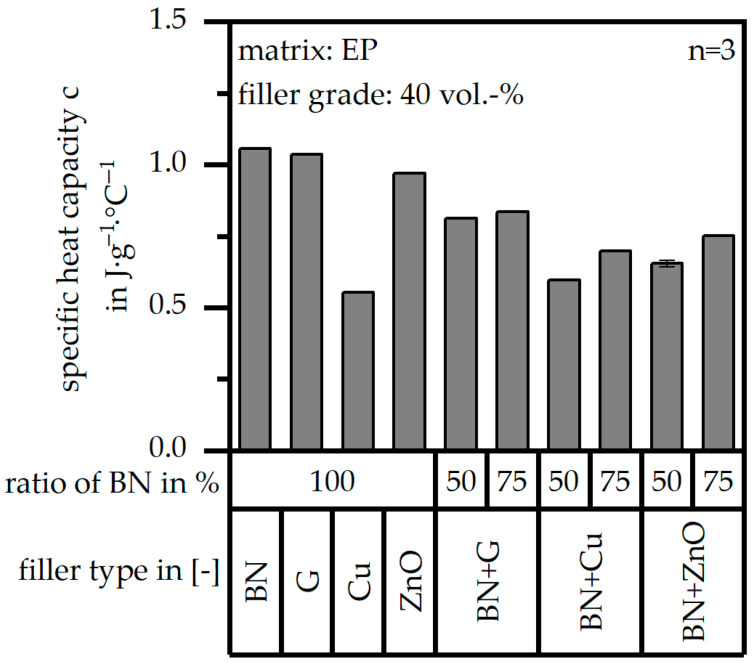
Specific heat capacity c of the compound material relative to the filler system (single or hybrid) with varying ratios (50:50 or 75:25) and a constant filler grade of 40 vol.-%.

The specific heat capacity c in terms of the single-filler and the hybrid filler systems is portrayed in Figure 4 with respect to different ratios within the hybrid ones. Copper (Cu) shows the lowest value for the specific heat capacity c within the single-filler systems, which goes along with the low value of the pure filler. However, zinc oxide (ZnO) depicts also a low value for the specific heat capacity c in the pure filler; but reaches similar values of c in the compound relative to boron nitride (BN) and glass (G). In the hybrid filler systems, the specific heat capacity c increases with the rising amount of boron nitride (BN), which shows the highest value of c in the pure filler except for that with glass (G). Therefore, this increasing effect is more notable in terms of copper (Cu) and zinc oxide (ZnO) in the hybrid system. Throughout the mixing of two different fillers, the range of the specific heat capacity c is reduced over the different materials, leading to similar behavior.

### 3.4. Results of Thermal Conductivity λ According to DIN EN ISO 22007

**Figure 5:**
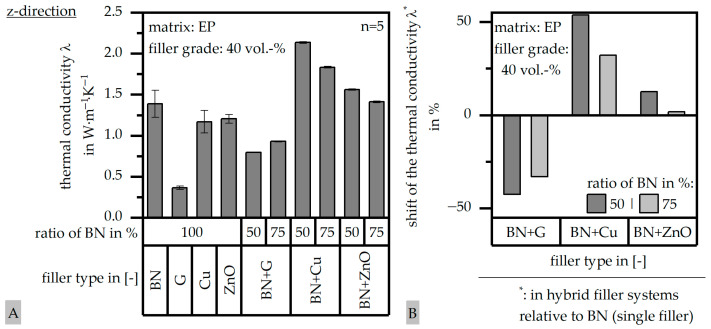
Thermal conductivity λ of the compound material relative to the filler system (single or hybrid) with varying ratios (50:50 or 75:25) and a constant filler grade of 40 vol.-%, (**A**) and a shift in the thermal conductivity λ in hybrid filler systems relative to boron nitride (BN) as the single filler (**B**).

The thermal conductivity λ in terms of the single-filler and the hybrid filler systems and relative to the different ratios is depicted in Figure 5A in terms of the z-direction (perpendicular to the flow direction). Further, the change in the thermal conductivity λ within the hybrid filler systems is shown in Figure 5B relative to the single-filler system with boron nitride (BN). With respect to the application in housings within power electronics, the thermal conductivity λ should reach at least a value of 10 W∙m^−1^∙K^−1^ [8] according to the literature. This value was not reached, which originates to some extent in the direction relative to the flow path and with the filler orientation, mainly in terms of the boron nitride. As shown in [27], the thermal conductivity λ increases by about 100% due to the change in direction (x-direction and parallel to the flow direction), 125% with a filler content of 60 vol.-%, and 100% with PF instead of EP as a matrix system. Taking all these factors, shown in [27], into account, the highest value in Figure 5, which is reached by a hybrid filler system based on boron nitride (BN) and copper (Cu) with a ratio of 50:50, could be theoretically extended to 16 W∙m^−1^∙K^−1^. Although this value was not proven within these experiments, once the focus is not placed on different material systems nor directions, the general potential of hybrid filler systems can be seen. Comparing the two spherical filler systems within the hybrid ones, it can be seen that the higher thermal conductivity λ of copper (Cu) as a pure filler massively increases the thermal conductivity λ in the hybrid filler system, whereas glass (G) reduces it due to its low thermal conductivity λ. Within a hybrid filler system, the combination of boron nitride (BN) and copper (Cu) reveals the potential of increasing the thermal conductivity λ by 50% compared to a single-filler systems with the same filler content in total. It can be seen that a lower amount of boron nitride (BN) with a maximum of 50% within the hybrid filler system has a greater impact on the overall increase in the thermal conductivity λ. This trend can also be seen in terms of zinc oxide (ZnO); nevertheless, the impact of zinc oxide (ZnO) is much lower than it is for copper (Cu).

### 3.5. Results of Mechanical Properties According to DIN EN ISO 527

In the following section, the results of the mechanical characterization are portrayed. Relative to [27], the results are compared to the values of the pure resin and of the industrial standard. Within electronic devices and the typical material system based on epoxy resin, the industry standards should reach the following values: an E-Modulus E_t_ of 17.400 MPa at least, a tensile strength σ_m_ of 77 MPa, and an elongation at break ε_m_ of 0.45%. The pure EP (type: 368/1) reveals the following values: an E-Modulus E_t_ of 4.400 MPa, a tensile strength σ_m_ of 12 MPa, and an elongation at break ε_m_ of 0.24%. These values are much smaller compared to the industrial standard and are further incomparable to the data given in the manufacturer’s specifications.

**Figure 6:**
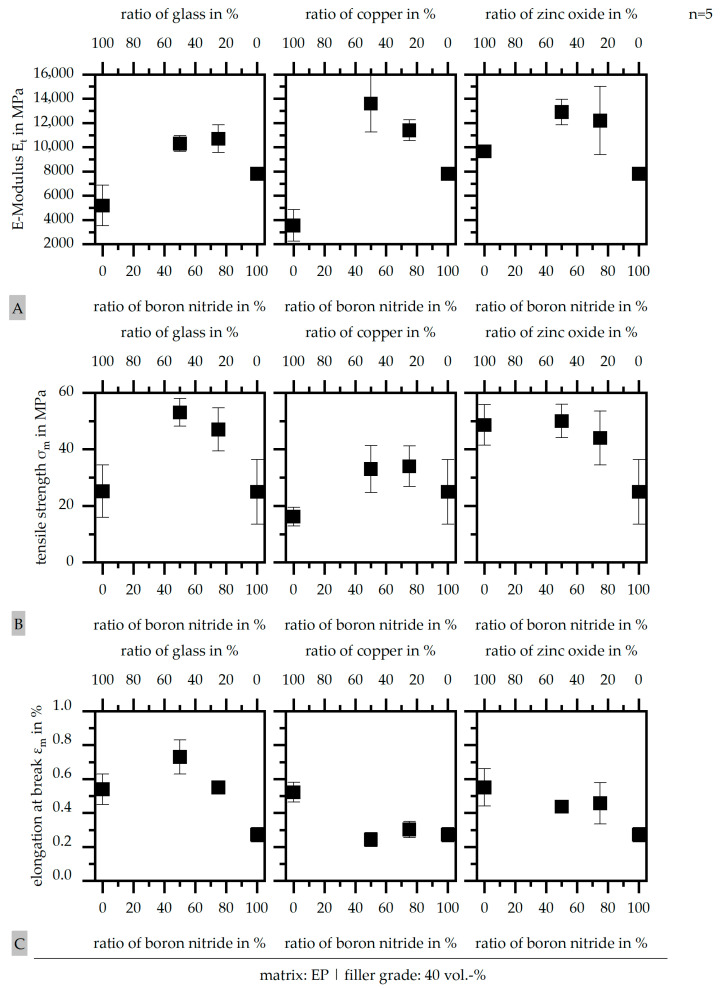
E-Modulus E_t_ (**A**), tensile strength σ_m_ (**B**), and elongation at break ε_m_ (**C**) of the compound materials relative to the filler system (single or hybrid) with varying ratios (50:50 or 75:25) and a constant filler grade of 40 vol.-%.

Figure 6 depicts the mechanical properties relative to the different filler systems and ratios (50:50 or 75:25) in comparison to the values for both single fillers. Figure 6A depicts the E-Modulus E_t_, which increases in terms of the hybrid filler system compared to all single-filler ones. The values are similar for the two different ratios, but reveal a synergistic effect by adding a second filler to boron nitride (BN). This improving effect reaches its maximum for copper (Cu) within these experiments with an increase of 75% compared to pure boron nitride (BN). Compared to the pure EP, the E-Modulus E_t_ is significantly improved by about 250% in terms of boron nitride (BN) and CU in a ratio of 50:50. However, the industrial standard is only 80% reached.

The tensile strength σ_m_ is shown in Figure 6B. Again, the values are increased in terms of the hybrid systems relative to the single-filler systems. The maximum is reached with glass as the second filler beside boron nitride (BN) with an increase of 150% compared to pure boron nitride (BN). In terms of zinc oxide (ZnO), the tensile strength σ_m_ is similar for pure zinc oxide (ZnO) and the hybrid systems, only with pure boron nitride (BN), a decrease is revealed. In general, copper (Cu) reaches a lower level compared to the other two fillers added to boron nitride (BN). All filler systems, independent of single-filler or hybrid ones, reach a higher tensile strength σ_m_ compared to pure EP, but they do not obtain the values of the industrial standard.

The elongation at break ε_m_ is shown in Figure 6C with a different behavior compared to the two other characteristic values of the mechanical properties. For the glass, the elongation at break ε_m_ again increases for the hybrid systems compared to the single-filler ones, reaching 250% higher values. For the copper (Cu) and zinc oxide (ZnO), the elongation at break ε_m_ decreases slightly for the hybrid systems with pure boron nitride (BN) compared to the pure second filler. Overall, the elongation at break ε_m_ of pure EP is reached for all systems and except for copper (Cu)-based material systems, where the industrial standard is exceeded.

### 3.6. Results of Filler Distribution

**Figure 7:**
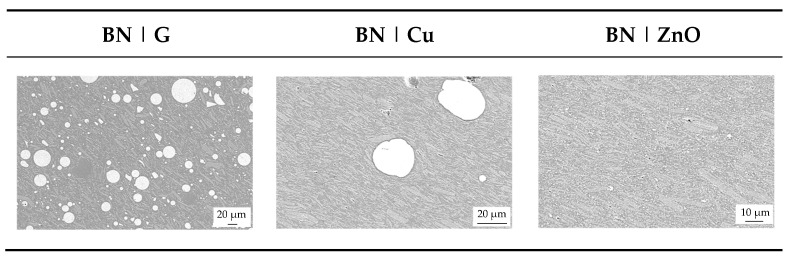
Image of the filler distribution and contact points of the compound materials relative to the filler systems (hybrid) with a ratio of 50:50 and a constant filler grade of 40 vol.-%.

The filler distribution and the contact zone between fillers in terms of hybrid systems are shown in Figure 7 exemplarily for the ratio of 50:50 for glass (G), copper (Cu), and zinc oxide (ZnO) in addition to boron nitride (BN). Zinc oxide (ZnO) does not reveal a star form anymore but rather is divided into many small parts. Due to this, the contact zone between boron nitride (BN) and zinc oxide (ZnO) is very small, which does not improve the thermal heat path through the sample to a high extent. Therefore, the thermal conductivity λ, as shown in Figure 5, does not increase significantly within the hybrid system with zinc oxide (ZnO). Copper (Cu), similar to glass (G), shows a higher contact zone, so the hybrid filler systems increase the thermal conductivity λ due to their improved thermal heat paths. Due to the large particle size of copper (Cu) compared to boron nitride (BN), the plate-like structure of boron nitride (BN) can align around the copper (Cu) spheres with direct contact. In can be assumed that within this pairing, even a higher amount of copper (Cu) would have an positive impact on the increase in the thermal conductivity λ.

## 4. Discussion

**Figure 8:**
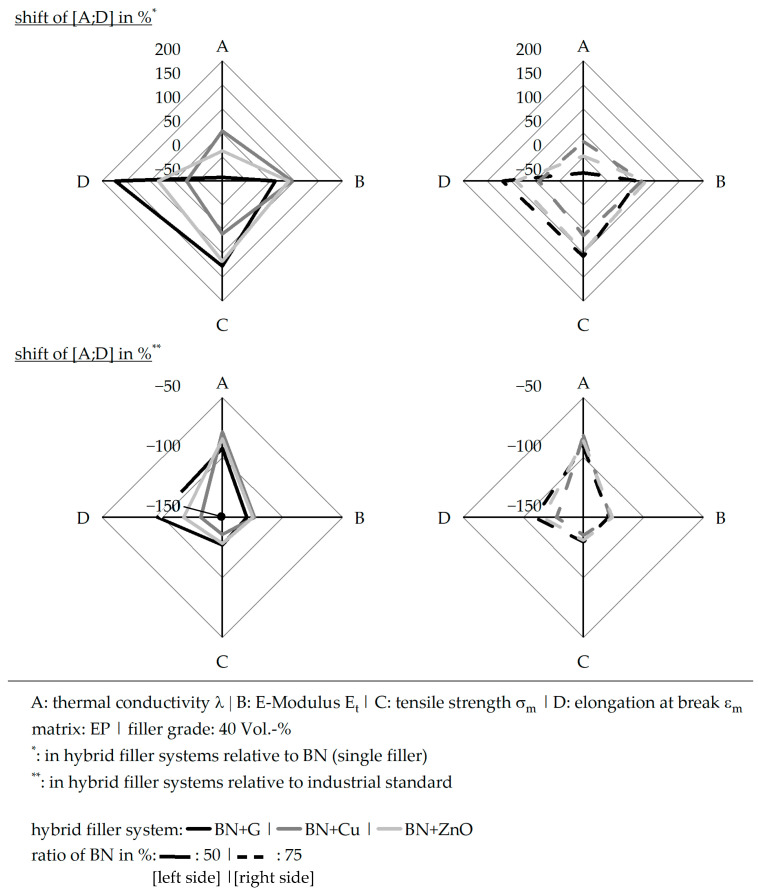
Comparison of the shifts in different characteristic values in terms of the thermal conductivity λ and the mechanical properties relative to pure boron nitride (BN) and the industrial standard for the compound materials based on hybrid filler systems with varying ratios (50:50 or 75:25) and a constant filler grade of 40 vol.-%.

The focus of these investigations was two-parted, seeking a high thermal conductivity λ and sufficient mechanical properties reached by hybrid filler systems. Furthermore, an adequate thermal flow path in terms of the thermal conductivity λ was aspired for. Within the experiments, boron nitride (BN) as the main filler was chosen mainly in terms of its high thermal conductivity λ, as shown in [27], for example. The second fillers were glass and copper (Cu), both in a sphere form, as well as ZnO. With that, the geometry of the filler was further evaluated in addition to the material type of the second filler in the hybrid system. Figure 8 depicts the changes in the main values (thermal conductivity λ, E-Modulus E_t_, tensile strength σ_m_, and the elongation at break ε_m_) compared to pure boron nitride (BN) and the industrial standard. It has to be taken into account that the thermal conductivity λ was only evaluated in the z-direction; it was discussed in Section 3.4 that a theoretical value of 16 W∙m^−1^∙K^−1^ can be reached. With that, copper (Cu) in the hybrid system depicts the best compromise between a high thermal conductivity λ and sufficient mechanical properties. The E-Modulus E_t_ of a hybrid system based on copper (Cu) with a ratio of 50:50 is highly increased; however, the increase in the tensile strength σ_m_ is lower compared to ZnO and not in terms of the elongation at break ε_m_. Especially in terms of the tensile strength σ_m_ and the elongation at break ε_m_, copper (Cu) and glass as second fillers behave highly differently. Since the free surface energies of copper (Cu) and glass are highly different and copper (Cu) is more similar to boron nitride (BN) than glass, not only the shape of the filler but rather the surface energy plays an important role in terms of the mechanical properties. Based on the experiments, the E-Modulus E_t_ is improved in a hybrid filler system, if the surface energy levels of both fillers are similar. The more they vary, the better the tensile strength σ_m_ and the elongation at break ε_m_ become. Furthermore, the shape of the fillers is important, as sphere shapes improve the mechanical properties in the hybrid systems compared to small fractures. In terms of the thermal conductivity λ, a high contact zone between the two fillers of the hybrid system should be reached. In terms of the platelet structure of boron nitride (BN), large spheres are likely to achieve this. Compared to the industrial standard, copper (Cu) as the second filler reveals the lowest values in terms of the elongation at break ε_m_. Since the filler grade within the experiments was quite low with 40 vol.-%, a further increase in mechanical supportive fillers could be evaluated as an option. In addition, the industrial standard should be reconsidered in terms of the new material systems and the actual application conditions.

## 5. Conclusions

Within this paper, three different fillers were evaluated with different second fillers in hybrid filler systems based on boron nitride (BN). The total filler grade was kept constant at 40 vol.-%, but the ratio between both fillers was varied as either 50:50 or 75:25. The aim of the investigation was the improvement of the thermal conductivity λ and the mechanical properties. It was shown that copper (Cu) as the second filler depicts the best compromise for this goal. Furthermore, criteria of the improvement of the thermal flow path and the mechanical properties were presented. Within them, similar surface energy levels of the different fillers are favorable in terms of the mechanical properties. The spherical form of one of the fillers further improved the mechanical properties. In terms of the flow path and the thermal conductivity, the combination of large spheres with smaller platelets (as was reached with boron nitride (BN) and copper (Cu)) allow for a high number of contact points with less interruption in the flow path.

## Figures and Tables

**Table 1:** Specification of the matrix material including density δ, heat capacity c (our own measurements), and thermal conductivity λ (manufacturer specification).

Matrix Material	Density δ in g∙cm^−3^	Heat Capacity c in J∙g^−1^∙°C^−1^	Thermal Conductivity λ in W∙m^−1^∙K^−1^
epoxy resin (EP)	1.2250	1.616	0.4–0.6

**Table 2:** Specifications of the filler materials including their density *δ*, particle size [n: numerical | v: volumetric counting], heat capacity c (our own measurements), and thermal conductivity λ (manufacturer’s specification).

Filler System	Density δ in g∙cm^−3^	Particle Size [n | v] in µm	Heat Capacity c in J∙g^−1^∙°C^−1^	Thermal Conductivity λ in W∙m^−1^∙K^−1^
boron nitride (BN)	2.27	4.77 | 24.34	0.794	15 ⊥; 400 ‖
glass (G)	2.48	4.70 | 18.53	0.808	1
copper (Cu)	8.65	18.19 | 41.60	0.394	400
zinc oxide (ZnO)	5.65	1.64 | 3.30	0.505	30

**Table 3:** Specifications of the filler systems including the particle geometry (based on our own measurements).

Filler System	BN	G	Cu	ZnO
**particle** **geometry**	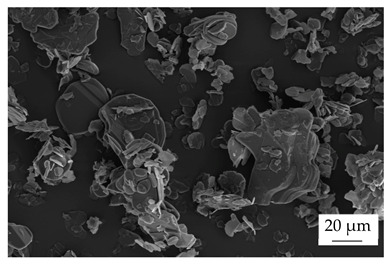	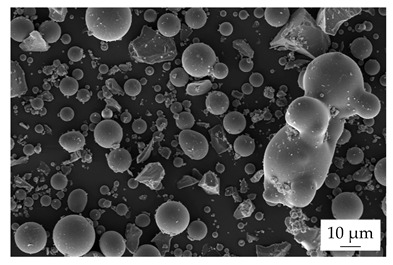	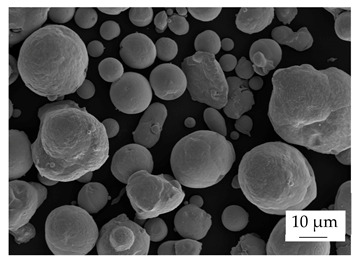	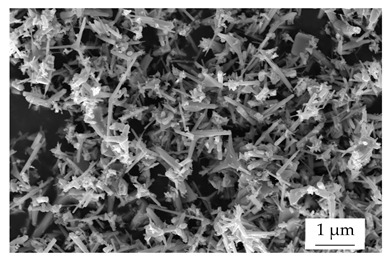
	platelet	sphere	sphere	star

**Table 4:** Process parameters of the fabricated test specimens relative to the material system.

Matrix Material	EP	EP	EP	EP	EP	EP	EP
filler system	BN	G	Cu	ZnO	BN + G	BN + Cu	BN + ZnO
filler grade	40 vol.-%						
mixing proportion	-				50 | 50 || 75| 25		
process parameter							
mass temperature T_m_ in °C [feeding | noozle]	55 | 95	55 | 85	65 | 85	65 | 85	55 | 85	55 | 85	55 | 85
mold temperature T_WZ_ in °C	160	195	180	180	190	190	190
heating time t_h_ in s	85	75	75	75	85	85	85
injection speed v_in_ in mm∙s^−1^	15	15	15	15	15	15	15

## Data Availability

Restrictions apply for the availability of these data. Data are available with the permission of the author.
